# Acute Arboviral Infections in Guinea, West Africa, 2006

**DOI:** 10.4269/ajtmh.2010.09-0688

**Published:** 2010-08-05

**Authors:** Emily S. Jentes, Jaimie Robinson, Barbara W. Johnson, Ibrahima Conde, Yosse Sakouvougui, Jennifer Iverson, Shanna Beecher, M. Alpha Bah, Fousseny Diakite, Mamadi Coulibaly, Daniel G. Bausch

**Affiliations:** Department of Tropical Medicine, Tulane School of Public Health and Tropical Medicine, New Orleans, Louisiana; Division of Vector-Borne Infectious Diseases, Centers for Disease Control and Prevention, Fort Collins, Colorado; Centre International de Recherche sur le Infections Tropicales, N'Zérékoré, Guinea

## Abstract

Acute febrile illnesses comprise the majority of the human disease burden in sub-Saharan Africa. We hypothesized that arboviruses comprised a considerable proportion of undiagnosed febrile illnesses in Guinea and sought to determine the frequency of arboviral disease in two hospitals there. Using a standard case definition, 47 suspected cases were detected in approximately 4 months. Immunoglobulin M antibody capture enzyme-linked immunosorbent assays and plaque-reduction neutralization assays revealed that 63% (30/47) of patients were infected with arboviruses, including 11 West Nile, 2 yellow fever, 1 dengue, 8 chikungunya, and 5 Tahyna infections. Except for yellow fever, these are the first reported cases of human disease from these viruses in Guinea and the first reported cases of symptomatic Tahyna infection in Africa. These results strongly suggest that arboviruses circulate and are common causes of disease in Guinea. Improving surveillance and laboratory capacity for arbovirus diagnoses will be integral to understanding the burden posed by these agents in the region.

## Introduction

Acute febrile illnesses comprise the majority of the disease burden to most populations in sub-Saharan Africa.^1^,^2^ Although a considerable percentage of these syndromes is attributable to familiar diseases such as malaria and typhoid fever, recent studies suggest that malaria may be overdiagnosed and that a significant proportion of febrile diseases may be caused by pathogens not frequently considered in most settings in sub-Saharan Africa.^3^–^6^

Arboviruses in West Africa include members of the genera *Flavivirus* [yellow fever (YFV), dengue (DENV), and West Nile (WNV)], *Alphavirus* [chikungunya (CHIKV) and O'nyong nyong (ONNV)], *Phlebovirus* [Rift Valley Fever (RVFV)], and *Bunyavirus* [Tahyna (TAHV)]. Evidence and reason suggest that these and other arboviruses circulate frequently in sub-Saharan Africa: competent mosquito vectors for many arboviruses are found throughout the region, the incidence of other mosquito-borne illnesses is generally high, and outbreaks of YFV, CHIKV, and other arboviruses are periodically noted. Whereas large outbreaks often initiate more active epidemiologic investigation and attempts at laboratory diagnosis, identification of arboviral syndromes on a daily basis in sub-Saharan Africa is rare. Detection is hampered by the non-specific clinical presentation, lack of local laboratory diagnostic facilities, cross-reactions on serologic testing, and passive and inconsistent surveillance in most African countries.^7^ The reported and confirmed cases of arbovirus infection are thought to represent only a small percentage of the actual cases.^8^,^9^ Without the possibility for laboratory confirmation, many clinicians' index of suspicion to diagnosis of arboviral syndromes is low.

**Figure 1.**
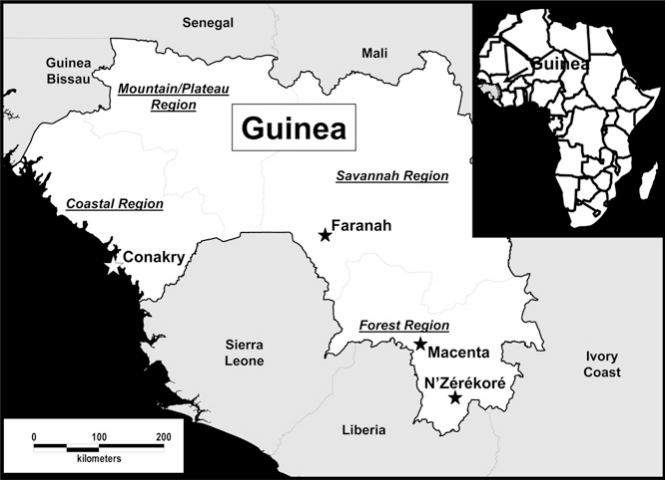
Guinea and surrounding countries in West Africa. The four defined topographical regions of Guinea are demarcated. Epicenters of YFV outbreaks in Guinea since 2000 are signified by stars. The study described here took place in N'Zérékoré (Forest Region) and Faranah (Savannah Region).

The Republic of Guinea is located on the Atlantic coast of West Africa, with a population of approximately 9.9 million. Evidence points to the circulation of various arboviruses in Guinea: outbreaks of YFV have occurred almost biannually since 2000 in various parts of the country, including northwestern Guinea (2000) and the towns of Conakry and N'Zérékoré (2001), Macenta (2003), and Faranah (2005) (Figure 1).^10^ The largest YFV outbreak reported in Africa was in Conakry in 2000–2001.^11^ A serosurvey of febrile patients between 1978 and 1989 found that 40% were positive for YFV by hemagglutination assay.^12^ Serological surveys have also showed antibodies to CHIKV in Guinea, although the morbidity associated with infection has not been thoroughly investigated^12^–^14^ (E. Jentes, unpublished data). Furthermore, a host of arboviruses have been isolated from arthropods, bats, birds, non-human primates, and rarely, humans in Guinea, although, with the exception of YFV, the incidence of human infection and disease due to arboviruses has not been reported nor systematically sought in that country.^12^,^13^,^15^–^22^ We hypothesized that arboviruses accounted for a considerable proportion of undiagnosed febrile illnesses in Guinea and sought to determine the frequency of human arboviral disease in two hospitals in two ecologically distinct regions of Guinea. The original intention was to conduct a matched case-control study over 1 year and establish diagnostics for arboviruses at the Center International de Recherche sur les Infections Tropicale in N'Zérékoré, Guinea. However, civil unrest, including nationwide strikes (including hospital staff), street violence, and gas shortages, forced closure of the study after just a few months. Nevertheless, the limited data that were collected and are reported here provide valuable information on the frequency and diversity of arbovirus infections in Guinea.

## Methods and Materials

### Study area.

Despite its abundant natural resources, including 30% of the world's bauxite reserves, Guinea remains one of the least developed countries in the world. Similar to many other sub-Saharan African nations, only 5.3% of the gross national product is spent on health. Agriculture, which is approximately 25% of the gross domestic product nationally, includes cultivation of rice, bananas, sweet potatoes, timber, and coffee. The average yearly temperature is 25.4°C, with average yearly rainfall of around 1,700 mm. There are single rainy and dry seasons extending from June to November and December to May, respectively.

Guinea is divided roughly into four distinct geographic and administrative regions: Basse Côte (lowlands near the oceanic coast), Fouta Djalon (mountainous middle region), Haute-Guinée (northeast savannah), and Guinée Forestière (southeastern rainforest). This study was conducted in the towns of N'Zérékoré and Faranah (Figure 1). Faranah (population of 88,000) is located in the savannah region (Haute-Guinée) along the Niger River basin, whereas N'Zérékoré (population of 120,000) is in an area of secondary tropical forest (Guinée Forestière) with interspersed agricultural plots. As in surrounding Liberia, Côte d'Ivoire, and Sierra Leone, Guinée Forestière has now been significantly deforested, leaving mainly palm and banana trees in areas that were previously dense with primary tropical forest.

### Human subject selection.

The study was approved by the Tulane University Internal Review Board and the Ethics Committee of the Guinean Ministry of Health. Patients who met a broad case definition for suspected acute febrile viral diseases were recruited from N'Zérékoré Regional Hospital (NZRH) and Faranah Regional Hospital (FRH) (Table 1). Patients were informed about the study, and oral consent was obtained before inclusion in the study. Because these hospitals charge for services on a sliding economic scale and are the reference centers for their respective regions, they are visited by people of all socio-economic levels from the town and surrounding prefecture and thus, have large catchment areas. NZRH has 175 beds, and FRH has 100 beds. Both hospitals provide services in general medicine as well as pediatric, maternity, and surgical wards and ambulatory services. Subjects were enrolled from August 2006 until civil unrest forced early termination of the study in January 2007. The study was initiated in August to correspond with the middle of the rainy season in Guinea, because the majority of cases of YFV in West Africa are noted from the middle of the rainy season to the middle of the dry season.^7^ The original intention of obtaining matched controls for each subject to investigate and compare risk factors for arbovirus infection in the community was not possible given the civil unrest.

### Specimen collection.

After explaining the objectives of the study and obtaining informed consent, 5 mL of blood were taken through venipuncture in red-top Vacutainer tubes (Becton, Dickinson, and Company, Franklin Lakes, NJ) from each subject at enrollment, which was usually on the first day of hospitalization (acute), and again on days 7 (late acute) and 28 after hospitalization (convalescent). However, for reasons of both subject adherence and civil unrest, we were often unable to obtain a complete set of three blood samples from each subject (see below). Because Lassa virus, a biosafety level-4 agent, is endemic in the area of study, all samples were inactivated by heating (56°C for 30 minutes) before further manipulation.^23^ This protocol has since been changed to 60°C for 60 minutes. The serum was separated from the clot by centrifugation and stored in labeled cryovials in a solar-powered freezer (approximately −11°C) until testing.

### Laboratory analysis.

All serum samples were first tested in Guinea by YFV immunoglobulin M (IgM) antibody capture (MAC) enzyme-linked immunosorbent assay (ELISA) and PanBio DEN IgM ELISA (PanBio Ltd, Brisbane, Australia) following the Centers of Disease Control and Prevention (CDC) Clinical Laboratory Improvement Amendments (CLIA)-approved protocol and manufacturer's instructions, respectively.^24^,^25^ When the deteriorating civil situation made it clear that the study must be terminated, the serum samples were transported on cold packs over land to Sierra Leone and subsequently, to Côte d'Ivoire, where they were shipped on dry ice to Tulane University in New Orleans, LA. Finally, they were shipped overnight to the Arboviral Diseases Branch of the CDC in Fort Collins, CO. At CDC, the samples were tested by MAC ELISA against a standard African antigen panel that includes WNV, YFV, DENV (serotype 2), CHIKV, and TAHV.^25^ Samples with positive or equivocal IgM results were confirmed by virus-specific neutralizing antibody titer 90% endpoint plaque-reduction neutralization assay (PRNT) in Vero cells using a 0.5% agarose double overlay visualized with neutral red staining in the second overlay.^26^,^27^ Neutralizing antibody titers were determined using PRNT 90% cutoff values for confirmation of IgM positive and equivocal samples.^28^,^29^ Neutralizing antibody titer is expressed as the reciprocal of the endpoint serum dilution that reduced the challenge virus plaque count by 90% based on the back titration. Samples were confirmed as positive with a positive or equivocal IgM and ≥ 4-fold neutralizing titer difference between paired specimens or between antibody titer to one virus over another, such as DENV and YFV. Those samples in which there was no neutralizing antibody titer or those that had less than a 4-fold difference in titer were classified as presumptive.

Data were recorded by hand on case-report forms and subsequently, were entered into electronic spreadsheets and imported into SPSS Statistics version 17.0 (SPSS, Inc., Chicago, IL) for statistical analysis. χ^2^, Yates' χ^2^, Fisher's exact, and Student's *t* tests were used as appropriate.

## Results

Forty-seven subjects were enrolled (Table 2). In 74% (35/47) of cases, only an acute sample was collected; in 37% (10/47), an acute and late acute sample were obtained, and in 4% (2/47), acute, late acute, and convalescent samples were obtained. There were no significant demographic differences between the patients enrolled at the two hospitals.

All 47 subjects tested negative for YFV or DENV infection by the MAC ELISAs performed in Guinea. However, based on the results of the tests conducted at CDC, arbovirus infections were considered confirmed or presumptive in 63% (30/47) of the subjects, 14 from N'Zérékoré and 16 from Faranah (Table 3). Infected patients were significantly younger than uninfected ones (33 versus 47 years, respectively; *P* = 0.009). Females (17/20; 85%) were more likely to be infected than males (13/27; 48%); *P* = 0.014); 14 (70%) of 20 women in the study were housewives, 13 (93%) of which were positive. There was no apparent geographic clustering of cases, with positives from 17 different villages (7 in and around N'Zérékoré and 10 in and around Faranah). Only 3 (10%) of 30 positive persons reported traveling outside their village in the 10 days before illness, suggesting that most, if not all, infections were acquired locally. Infections seemed to be roughly evenly dispersed across the various ethnic groups studied, with cases in 10 of 12 different groups represented in the study (Table 2). There was no association with marital status or the number of people living in the house.

Flavivirus infections, especially WNV, predominated, but infections were noted with viruses from all three genera tested (Table 3). In three cases, there was evidence of coinfection by two arboviruses: one TAHV/CHIKV, one WNV/CHIKV, and one CHIKV with another untypeable bunyavirus (this patient was positive on the ELISA IgM for Tahyna virus but was PRNT negative, presumably indicating infection with a different bunyavirus). No samples showed evidence of coinfection by viruses of the same genus.

The civil unrest often prevented physicians from coming to the hospital to make detailed clinical observations. Additionally, dates of onset were not reliably recorded. However, we were able to record the patients' self-reported symptoms, which tended to be very non-specific (Table 4). There were no statistical differences in symptoms between patients with and without arbovirus infection or between patients with specific arbovirus infections, although the small sample size in each virus category precluded meaningful statistical analysis. Vital signs taken on admission did not vary significantly between those with and without arbovirus infection, with the exception of the temperature (38.1°C and 37.3°C, respectively; *P* = 0.002). One patient with confirmed YFV infection reported a spontaneous abortion at home before coming to the hospital. Two patients died: one positive for DENV and the other with a presumptive diagnosis of TAHV infection.

## Discussion

Despite the brief surveillance period and unanticipated logistical impediments encountered in our study, over 60% of the patients that we sampled in two ecologically distinct regions of Guinea had evidence of arboviral syndromes. Five distinct arbovirus infections were noted. With the exception of yellow fever, these are the first reported cases of human disease caused by these viruses in Guinea to our knowledge. These results strongly suggest that various arboviruses circulate, are common causes of human disease in Guinea, and are likely to be markedly underdiagnosed.

Perhaps the least surprising finding of our study is identification of cases of YFV as YFV is endemic in sub-Saharan Africa.^10^ Despite a safe and effective vaccine, cases of YFV infection were reported in 13 of 14 West African countries at risk from 2000 to 2006, including numerous outbreaks in Guinea (Figure 1), although there was no recognized outbreak of YFV or any other arbovirus in Guinea during the period of our study.^10^,^11^,^30^,^31^ It should be noted that only two of the subjects in our study reported having received the YFV vaccine—one CHIKV case from FRH and one case who was negative for all viruses tested. Ten subjects reported never receiving YFV vaccine. Thirty-five subjects did not know their vaccination status, including the two positive for anti-YFV IgM antibody. The resurgence of YFV in West Africa is partially attributable to the end of the routine preventative immunization campaigns common during the colonial era, which ended in the late 1950s/early 1960s; however, many African countries, including Guinea, have added YFV vaccine to the recommended list of routine childhood vaccines.^30^

Our finding of two cases of YFV during such a brief period of more intensive surveillance suggests that YFV infection may be more common that typically noted or assumed. We suspect that more thorough surveillance, at least in rural areas, would show a baseline incidence of YFV infection that is > 0. Such a finding would have significant consequences given the present policy of implementing costly and labor-intensive mass vaccination campaigns based on the confirmation of a single case. Declaration of an outbreak should ideally take into account the level of baseline transmission, but such an approach will require markedly enhancing surveillance and laboratory capacity.

CHIKV infection was frequent in our study (17% of cases), a finding in keeping with previous reports from Guinea showing a seroprevalence of CHIKV antibody over 50%^13^ (E. Jentes, unpublished data). There are also numerous reports of CHIKV isolations from various arthropods and small mammals in Guinea.^12^,^15^,^32^ The geographic distribution of CHIKV virus includes most of sub-Saharan Africa, although the virus has not frequently been the subject of study in West Africa.^33^–^36^ CHIKV is considered endemic in most rural areas, with small numbers of cases occurring each year, whereas large and explosive periodic outbreaks may occur in urban areas.^13^,^34^,^37^–^40^ The finding of anti-CHIKV antibodies in 30–100% of the population in some studies suggests asymptomatic or mild infection to be frequent, although underdeveloped surveillance systems to detect and diagnose cases most certainly contribute to this finding and interpretation.^34^,^35^

We found one confirmed case of DENV in Guinea, the first human case to be reported from that country, although DENV-2 was isolated from a mosquito in 1996.^12^ The volume of serum was insufficient to perform further testing to identify the specific infecting DENV serotype. Further evidence that DENV circulates in Guinea comes from a 1-month pilot study that we conducted in N'Zérékoré in 2004 in which 3 of 13 (23%) febrile patients tested positive for anti-DENV IgM antibody, whereas IgG antibody to DENV was found in 34 of 261 (13%) convenience samples using the PanBio assays. Furthermore, only two of eight (25%) persons in the study known to be previously vaccinated against YFV tested IgG antibody positive for DENV, suggesting that YFV cross-reaction did not account for the majority of the positive results.

DENV-1, -2, and -4 have been repeatedly isolated from humans and mosquitoes in various countries of West Africa, including Nigeria, Senegal, and Côte d'Ivoire, although DENV-3 has been recently reported in Cape Verde and isolated in travelers returning from Senegal and Côte d'Ivoire.^41^–^51^ Reports of cases of dengue fever in Africa have increased over the past two decades, although the dramatic epidemics seen in other parts of the world have not occurred.^52^,^53^ All four serotypes have been found in sub-Saharan Africa, although dengue hemorrhagic fever has only been reported in a few isolated cases.^52^,^53^ Recently, it has been proposed that African sylvatic DENV strains are less virulent than strains circulating in other parts of the world and therefore, would not cause severe human disease.^54^,^55^

WNV was the most common arbovirus infection noted in our study. WNV was reportedly isolated from a wild rodent in Guinea in 2006, but this is the first report of human infection.^32^ Serological evidence of WNV infection in humans, horses, birds, and arthropods has been reported in neighboring Senegal and Cote d'Ivoire as well as in one confirmed case in a traveler returning from Senegal.^56^–^60^

TAHV is widely distributed in Europe and Asia but has not been thoroughly investigated in Africa. Serological evidence of human infection in Cameroon and small- mammal infection in Tunisia^62^ has been reported.^61^ To our knowledge, this is the first report of symptomatic TAHV infection in Africa.

Our study had several limitations. The various impediments imposed by the civil unrest in Guinea are described above. The possibility that the blood samples could contain Lassa virus necessitated heat inactivation, precluding any attempts at virus isolation, the gold standard and definitive evidence of arbovirus infection, or polymerase chain reaction (PCR). Frequent power outages posed a challenge to maintaining the cold chain, properly storing specimens and reagents, and performing the onsite serological tests. This perhaps accounts for the discrepancy between the negative findings in Guinea and the frequent positives found at CDC. Furthermore, the circuitous shipment of the serum specimens to the United States through Sierra Leone and Cote d'Ivoire resulted in numerous breaks in the cold chain, which ultimately precluded all but serological diagnosis and could have even resulted in deterioration of antibodies measured through these tests. However, if that were the case, our results represent an underestimate of the incidence of arbovirus infections in Guinea. It must also be noted that cross-reactions are always a potential issue in the serological diagnosis of arbovirus infections, although the conservative interpretation of the PRNTs performed in this study should limit any undue conclusions regarding the specific virus infections. Furthermore, even if cross-reactions result in errors identifying the specific infecting virus, the important conclusion that many arboviruses are circulating in the area remains valid. Lastly, we were not able to make detailed clinical observations of the patients, test for coinfections, or completely exclude the possibility of drug-resistant malarial or bacterial infections.

The results of our study should serve as a reminder to clinicians in Guinea that arboviruses are a frequent cause of febrile disease in that country and likely, the rest of West Africa as well. More intensive surveillance systems are needed to define the scope of the problem, but it can safely be assumed to be large. Improving laboratory capacity in sub-Saharan Africa for the diagnosis of arbovirus infections will be integral to a full understanding of the burden posed by these agents.

## Figures and Tables

**Table 1:** Case definition used to detect patients with suspected acute febrile viral diseases at N'Zérékoré and Faranah Regional Hospitals*

Major signs	Minor signs
Abnormal bleeding (from the mouth, nose, rectum, and/or vagina)	General malaise
Edema of the neck and/or face	Headache
Conjunctival or sub-conjunctival hemorrhage	Retrosternal pain
Jaundice	Muscle or joint pain
Spontaneous obortion	Vomiting
Buzzing in the ears or acute deafness	Cough
Persistent hypotension	Sore throat
Elevated liver transaminases (serum glutamic oxaloacetic transaminase [SGOT]/aspartate aminotransferase [AST])	Abdominal pain
	Diarrhea
	Proteinuria
	Leucopenia < 4,000/μL

* For inclusion in the study, the patient must present with fever > 38°C for less than 3 weeks, absence of signs of local inflammation (i.e., the illness is systemic), negative thick smear for malaria, absence of a clinical response after 48 hours of antimalaria treatment and/or broad-spectrum antibiotics, and two major signs or one major sign and two minor signs. Common antimalarial drugs used in the area include chloroquine, quinine, sulfadoxine/pyrimethamine, and artemisinin compounds. Common antibiotic regimens include combinations of various beta-lactams, including penicillin derivatives and cephalosporins, aminoglycosides, sulfa drugs, macrolides, and chloramphenicol.

**Table 2:** Demographic findings of 47 patients presenting with suspected acute febrile viral diseases to N'Zérékoré (NZRH) and Faranah (FRH) Regional Hospitals [*n* (column %)]*

Characteristic	Hospital		Total
	NZRH	FRH	
Number of subjects enrolled	24	23	47
Sex†			
Male	15 (63)	12 (52)	27 (57)
Female	9 (37)	11 (48)	20 (43)
Age (years)†			
≤ 20	6 (25)	1 (4)	7 (15)
21–40	12 (50)	14 (61)	26 (55)
41–60	3 (12.5)	5 (22)	8 (17)
≥ 61	3 (12.5)	3 (13)	5 (13)
Profession†			
Housewife	7 (29)	7 (30)	14 (30)
Farmer	4 (17)	9 (39)	13 (28)
Merchant	5 (21)	0 (0)	5 (11)
Student	4 (17)	0 (0)	4 (9)
Driver	2 (8)	1 (4)	3 (6)
Other	2 (8)	6 (26)	8 (17)
Ethnicity‡			
Malinké	8 (35)	11 (48)	19 (41)
Peuhl	3 (13)	6 (26)	9 (20)
Konianké	5 (22)	0 (0)	5 (11)
Guerzé	3 (13)	1 (4)	4 (9)
Kissi	1 (4)	1 (4)	2 (4)
Soussou	1 (4)	0 (4)	1 (2)
Other	2 (9)	4 (17)	6 (13)

NZRH = N'Zérékoré Regional Hospital; FRH = Faranah Regional Hospital.

* Percentages may not add up to 100% because of rounding.

† There was no significant difference in the sexes (χ^2^ = 0.5, *P* = 0.47), ages (*t* test = 0.13, *P* = 0.90), professions (housewife, farmer vs. all other categories; χ^2^ = 3.7, *P* = 0.16), and ethnicities (Malinké, Peuhl vs. all other categories; Yates' χ^2^ = 2.0, *P* = 0.36) of patients between NZRH and FRH. Categories were collapsed for statistical comparison because of small sample size; however, all categories are shown in the table for descriptive purposes.

‡ Ethnicity missing for one patient from NZRH.

**Table 3:** Results of ELISA and PRNTs for a standard panel of African arboviruses on 47 patients presenting with suspected acute febrile viral diseases to N'Zérékoré Regional Hospital (NZRH) and Faranah Regional Hospital (FRH)

Virus	Hospital		Total* (% of total)
	NZRH (% tested)*	FRH (% tested)*	
Flaviviruses			
Yellow fever			
Confirmed†	0 (0)	1 (4)	1 (2)
Presumptive†	0 (0)	1 (4)	1 (2)
West Nile			
Confirmed	1 (4)	1 (4)	2 (4)
Presumptive	8 (33)	1 (4)	9 (19)
Dengue			
Confirmed	0 (0)	1 (4)	1 (2)
Presumptive	0 (0)	0 (0)	0 (0)
Alphaviruses			
Chikungunya			
Confirmed	1 (4)	3 (13)	4 (9)
Presumptive	1 (4)	3 (13)	4 (9)
Bunyaviruses			
Tahyna			
Confirmed	1 (4)	0 (0)	1 (2)
Presumptive	2 (8)	2 (9)	4 (9)
Multiple infections			
Tahyna confirmed/chikungunya presumptive	0 (0)	1 (4)	1 (2)
West Nile confirmed/chikungunya presumptive	1 (4)	0 (0)	1 (2)
Chikungunya confirmed/presumptive non-Tahyna bunyavirus	1 (4)	0 (0)	1 (2)
Negative	8 (33)	9 (39)	17 (36)
Total	24	23	47

* Percentages may not add up to 100% because of rounding.

† See Methods and Materials for definition of confirmed and presumptive cases.

**Table 4:** Self-reported symptoms in patients with suspected arbovirus infections presenting to N'Zérékoré and Faranah Regional Hospitals

Symptom	Number reporting (%)					
	All patients	Positive for any virus*	Flavivirus positive†		Alphavirus positive*	Bunyavirus positive*
			YFV	WNV	CHIKV	TAHV
Fever‡	42/44 (96)	26/28 (93)	2/2 (100)	11/11 (100)	11/11 (100)	4/5 (80)
Malaise	42/44 (96)	26/28 (93)	0/1 (−)	12/12 (100)	10/11 (91)	6/6 (100)
Headache	38/45 (84)	23/29 (79)	0/1 (−)	10/12 (83)	11/11 (100)	4/6 (67)
Light-headedness	35/44 (80)	20/28 (71)	0/1 (−)	9/12 (75)	9/10 (90)	4/6 (67)
Muscle aches	34/44 (77)	22/28 (79)	1/1 (100)	11/12 (92)	7/11 (64)	4/6 (67)
Nausea/vomiting	34/45 (76)	23/28 (82)	1/2 (50)	10/12 (83)	9/10 (90)	5/6 (83)
Abdominal pain	30/42 (71)	18/25 (72)	2/2 (100)	9/12 (75)	5/7 (71)	4/6 (67)
Joint pain	30/44 (68)	19/28 (68)	0/1 (−)	10/12 (83)	6/11 (55)	5/6 (83)
Back pain	29/44 (66)	19/28 (68)	0/1 (−)	9/12 (75)	8/11 (73)	3/6 (50)
Cough	26/42 (62)	17/26 (65)	1/1 (100)	8/11 (73)	7/10 (70)	2/6 (33)
Shortness of breath	27/44 (61)	17/27 (63)	1/1 (100)	9/12 (75)	6/9 (67)	4/6 (67)
Chest pain	24/42 (57)	17/26 (65)	1/1 (100)	9/12 (75)	5/9 (56)	3/6 (50)
Epigastric pain	24/43 (56)	15/26 (58)	1/2 (50)	8/12 (67)	5/8 (63)	2/6 (33)
Diarrhea	23/44 (52)	14/27 (52)	2/2 (100)	8/12 (67)	3/9 (33)	3/6 (50)
Yellow eyes/jaundice	17/36 (47)	12/23 (52)	0/1 (−)	4/10 (40)	5/9 (56)	3/5 (60)
Ringing in the ears	14/40 (35)	9/25 (36)	0/1 (−)	5/10 (50)	4/10 (40)	1/6 (17)
Sore throat	13/39 (33)	8/23 (35)	0/1 (−)	5/10 (50)	2/8 (25)	2/6 (50)
Nasal congestion	12/41 (29)	9/25 (36)	0/1 (−)	7/12 (58)	2/8 (25)	1/6 (17)
Gum/oral bleeding	10/44 (23)	8/26 (31)	2/2 (100)	5/12 (42)	1/9 (11)	1/6 (17)
Red eyes/conjunctivitis	7/39 (18)	6/23 (26)	0/1 (−)	3/10 (30)	2/8 (25)	1/6 (17)
Facial or neck swelling	7/43 (16)	6/27 (22)	0/1 (−)	40/11 (36)	2/11 (18)	1/6 (17)
Vomiting blood/hematemesis	7/44 (16)	6/27 (22)	1/2 (50)	1/12 (8)	3/9 (33)	1/6 (17)
Nose bleeds/epistaxis	7/43 (16)	5/26 (19)	2/2 (100)	3/12 (25)	2/8 (25)	0/6 (−)
Bloody or black stools/melena	6/44 (14)	4/27 (15)	1/2 (50)	1/12 (8)	1/9 (11)	1/6 (17)
Mouth ulcers	4/42 (10)	3/26 (12)	0/1 (−)	2/11 (18)	2/10 (20)	0/6 (−)
Hearing loss	4/42 (10)	2/22 (9)	0/1 (−)	2/9 (22)	0/8 (−)	0/6 (−)
Swollen lymph nodes	3/43 (7)	3/27 (11)	0/1 (−)	3/11 (27)	1/11 (9)	0/6 (−)
Rash	2/41 (5)	2/25 (8)	0/1 (−)	2/10 (20)	1/10 (10)	0/6 (−)
Vaginal bleeding	2/41 (5)	2/26 (8)	1/2 (50)§	1/12 (8)	0/8 (−)	0/6 (−)
Bloody urine	1/42 (2)	1/26 (4)	1/2 (50)	0/11 (−)	0/9 (−)	0/6 (−)

CHIKV = chikungunya virus; DENV = dengue virus; TAHV = Tahyna virus; WNV = West Nile virus; YFV = yellow fever virus.

* Includes both confirmed and presumptive cases.

† The one person positive for DENV was unconscious on admission and subsequently died. No information was available on symptoms at disease onset.

‡ In two cases, patients reported fever > 38°C for less than 3 weeks but were afebrile on presentation to the hospital.

§ The patient reported a spontaneous abortion before coming to the hospital.
