# Erratum

**DOI:** 10.4269/ajtmh.2010.835err

**Published:** 2010-11-05

In *Am J Trop Med Hyg 83:* 388–394 by Jentes et al., one of the author's names was inadvertently omitted. Juliet Bryant, Vector-Borne Infectious Diseases, Centers for Disease Control and Prevention, Fort Collins, Colorado, should be listed as a co-author on this manuscript.

